# Supplementary material for: Multi‐dataset identification of innovative feature genes and molecular mechanisms in keratoconus
Source: J Cell Mol Med. 2024 Sep 19;28(18):e70079. doi: 10.1111/jcmm.70079 (PMC11412914; doi:10.1111/jcmm.70079)
Supplement: Supplementary file 2 — Material S2. [file JCMM-28-e70079-s002.zip › 7.GSEA analysis/2.ATOH7/heat_map_corr_plot.html]

Heat map and correlation plot for ATOH7  

Fig 1: heat\_map      
 Heat Map of the top 50 features for each phenotype in ATOH7

  
  

Fig 2: Ranked Gene List Correlation Profile      
 Ranked list correlations for ATOH7

  
  
    
